# Post Traumatic Endophthalmitis: Incidence and Risk Factors

**DOI:** 10.5539/gjhs.v6n6p68

**Published:** 2014-06-30

**Authors:** Ali Reza Dehghani, Leila Rezaei, Hasan Salam, Zahra Mohammadi, Mohammad Mahboubi

**Affiliations:** 1 Ophthalmology Isfahan University of Medical Sciences, Isfahan, Iran; 2 Ophthalmology Kermanshah University of Medical Sciences, Kermanshah, Iran; 3 Health Services Administration, Kermanshah University of Medical Sciences, Kermanshah, Iran; 4 Abadan School of Medical Sciences, Abadan, Iran

**Keywords:** endophthalmitis, Isfahan, traumatic

## Abstract

Post traumatic endophthalmitis is an uncommon but severe complication of ocular trauma. We aimed to identify the incidence of post traumatic endophthalmitis and its contributing risk factors in Feiz hospital (Isfahan, Iran) from 2006 until 2010. Medical records of 1042 patients with open globe injury were analyzed and data were collected including age, sex, location of being injured, visual acuity (VA), time from injury to hospitalization and to repair, site of ophthalmic injury and the presence of foreign body. The frequency of post-traumatic endophthalmitis was about 2.1% (N = 22) of all patients. Nine of 22 cases with endophthalmitis were under 8 years. The visual acuity at the time of admission was seen to be contributed to high rate of endophthalmitis. Intraocular foreign body was detected in 139 patients; and the rate of endophthalmitis was 5% among these patients. Statistical analysis showed significant relationship between presence of foreign body and higher rate of endophthalmitis. Also, duration of hospitalization was significantly different between two study groups (P = 0.019). There were no significant differences between two groups in terms of other studied variables. Patients with low age, low visual acuity at admission, presence of intraocular foreign body and long duration of hospital stay had a higher risk of endophthalmitis after the repair of the globe. Compared to the reports of other large institutions, we can attribute the low incidence rate of endophthalmitis in our institution to the early use of systemic antibiotics such as gentamycin and cephalosporins in the first hour of hospitalization until discharge.

## 1. Introduction

Post traumatic endophthalmitis still remains an uncommon but a serious and somewhat devastating complication after repairing of open globe injury. The incidence of post traumatic endophthalmitis varies from 3.3% to 17% in series from large institutions. The likelihood of infection after penetrating trauma surgery is approximately 100 times greater than post elective cataract surgery (Meredith, 1999). Ocular signs associated with post traumatic endophthalmitis are similar to those with post-operative infections, including anterior chamber (AC) cell and flare, hypopyon, and vitreous cell exceeding that expected from the injury itself (Schoenberger, 2012). It is associated with special spectrum of organisms such as *Bacillus*, *staphylococcus* and *streptococcus* species (Realini, 2007) and is generally associated with a poor outcome despite recent advances in vitreoretinal techniques and intraocular antibiotic use (Boldt, 1989).

Some factors are reported to be associated with increased risk of post traumatic endophthalmitis, including retained intraocular foreign body, lens rupture, delayed timing of primary repair, large wound size, location of wound, ocular tissue prolapsed, age greater than 50 years, female gender, and rural location of being injured (Andreoli et al., 2009; Brinton et al., 1984; Essex, Charles, & Allen, 2004).

For initial therapy, some studies recommended empiric antibiotics for all cases of traumatic endophthalmitis which are vancomycin and ceftazidim administered as intravitreal injections (Bhagat, Nagori, & Zarbin, 2011), whereas others encourage early deep vitrectomy (Danis, 2002).

In this study we aimed to identify the incidence of post traumatic endophthalmitis and the related risk factors influencing its development.

## 2. Method

In this retrospective descriptive analysis, the patients who were refered to Feiz hospital (Isfahan, Iran) from 2006 until 2010 were included. Medical records of patients with open globe injury were analyzed and data were collected including age, sex, location of being injured, visual acuity (VA), time from injury to hospitalization and to repair, site of ophthalmic injury and the presence of foreign body. The exclusion criterion was enucleation.

### 2.1 Pre- and Post-Operative Examinations and Interventions

As the patients were referred to the emergency department, they were undergone examinations including assessment of visual acuity and relative afferent pupillary defect (RAPD), determining type of injury, X-ray examination of orbit, full slit lamp examination, and eye shield. After hospitalization, intravenous Keflin 50 mg/kg/day divided in four doses and Gentamicin 1 mg/kg/day divided in three doses were started in the first hour, as soon as the injury was repaired using the standard techniques. The patients then were observed to any progress in the clinical signs. Ciprofloxacin and betamethasone eye drops were prescribed to the patients, every three and six hours, respectively.

In our study, endophthalmitis was defined as any deterioration in the clinical signs such as hypopyon, decline in the VA, progression in marcus gan, purulent discharge and positive microbial culture, although the decision for the treatment is taken due to the clinical signs and not waiting for culture. The rural trauma was defined for any trauma that happens in the garden, farm or typical rural homes. All of detected endophthalmitis cases had undergone deep vitrectomy with or without silicone injection.

### 2.2 Statistical Analysis

Data was analyzed with SPSS software version 20, using T-test, Chi-square or Fisher exact test for variable comparisons between groups. P value less than 0.05 was considered statistically significant.

## 3. Results

We analyzed medical records of 1042 patients with penetrating globe injury who were admitted to the emergency department of Feiz Hospital, Isfahan, Iran. The overall frequency of post-traumatic endophthalmitis was about 2.1% (N=22) of all patients. Demographic data including age and sex, elapsed time since trauma to hospitalization and since trauma to the repair of injury, duration of hospitalization, and place of being injured (rural or urban) are presented in Table 1.

**Table 1:** Demographic and overall data of the study population

Variable	Patients with endophthalmitis (N=22)	Patients without endophthalmitis (N=1020)	*P*-value*
Sex			
Male	19 (86%)	818 (80%)	0.344
Female	3 (14%)	202 (20%)	

Place of being injured			
Rural	16 (73%)	773 (76%)	0.452
Urban	6 (27%)	247 (24%)

Age (years)	19.2 ± 16.7 (Range: 3.5 - 62)	24.8 ± 17.7 (Range: 1 - 94)	0.04*
Time from trauma to hospitalization (hours)	19 ± 28.8	21 ± 52.8	0.87
Time from trauma to repair of injury (hours)	25 ± 28.5	26 ± 48.7	0.94
Duration of hospitalization (days)	4.7 ± 1.9	3.8 ± 1.5	0.009*

Data are presented as mean ± SD or N (%), where applicable.

*P*-value

* <0.005.

Clinical data including visual acuity, presence of foreign body, and site of laceration are presented in Table 2. The visual acuity at the time of admission was seen to be contributed to high rate of endophthalmitis. In our study, all of 22 cases with endophthalmitis had VA less than 1/10 snellen charts on admission, and none of the patients with VA more than 1/10 have been involved with endophthalmitis.

**Table 2:** Clinical data of the study population

Variable	Patients with endophthalmitis (N=22)	Patients without endophthalmitis (N=1020)	*P*-value
Visual acuity (VA)			
No light perception	4 (19%)	99 (9.8%)	-
Light perception	6 (27%)	200 (19.6%)	
Hand motion	6 (27%)	214 (20.9%)	
Finger count	6 (27%)	205 (20.1%)	
> 1/10 Snellen	0 (0%)	212 (20.8%)	
Undetermined	0 (0%)	90 (8.8%)	

Presence of foreign body	7 (32%)	132 (12.9%)	0.019*

Site of laceration			
Cornea	12 (54.5%)	444 (43.5%)	0.174
Sclera	7 (31.8%)	246 (24%)	0.132
Sclerocornea	3 (13.7%)	330 (32.5%)	0.110

Data are presented as N (%).

* Analysis with Fisher exact test.

Intraocular foreign body was detected in 139 patients; and the rate of endophthalmitis was 5% among these patients. Analysis with Fisher exact test showed statistically significant relationship between presence of foreign body and higher rate of endophthalmitis (P = 0.019).

The site of trauma was also investigated with regard to its relation to higher rate of endophthalmitis. Three sites were determined: cornea, sclera and sclerocornea; regarding these sites, the rate of endophthalmitis was 2.6%, 2.8% and 0.9%, respectively. No statistically significant difference was seen after analysis with Chi-square test. Also, there were significant differences between two groups regarding duration of hospitalization. Other variables did not differ significantly between groups.

Results of the microbial culture from the vitreous of involved eyes revealed only nine contaminated cases as following: three patients contaminated with *Bacillus* spp., two patients contaminated with *Staphylococcus aureus*, three patients contaminated with *Streptococcus* spp. and one patient contaminated with *Staphylococcus* *epidermidis*; other cultures were negative.

## 4. Discussion

The post traumatic endophthalmitis is still one of the important complications after the repair of open globe injury. In this study, our goal was to investigate the incidence and the contributing risk factors of endophthalmitis to make the correct decision in the management of such clinical challenge in high risk patients to decrease the incidence and the sequel of this devastating event.

The overall incidence of post-traumatic endophthalmitis in our study was 2.1% which was clearly less than findings (3.3% to 17%) reported from other large institutions (Meredith, 1999). The incidence rate of endophthalmitis was reported to be 3.4% by United States eye registry in 2002 (Danis, 2002). In a large Iranian study by Faghihi et al., the rate of endophthalmitis was 5.1% in patients with open-globe injury (Faghihi et al, 2012). Some studies reported low incidence of endophthalmitis (1% to 7%) and attributed this to the use of systemic antibiotics (Charles, 2003). We also can attribute the low incidence rate of endophthalmitis in our institution to the early use of systemic antibiotics such as gentamycin and cephalosporins in the first hour of hospitalization until discharge.

The low age of the patients was found to be associated with higher rate of endophthalmitis. Nine of 22 cases with endophthalmitis were under 8 years. It seems that younger patients have less compliance for treatment and they do not cooperate with the examiner when they are under observation due to the pain after repair. This behavior can result in delaying diagnosis and treatment (Narang et al., 2004).

In one study, post-traumatic endophthalmitis developed in 14% of patients under 15 years. One study reported that trauma was the cause of endophthalmitis in 86% of cases in pediatric population.

The presence of intraocular foreign body has contributed to higher rate of endophthalmitis in many studies (Hosseini et al., 2011); in our study, the incidence of endophthalmitis was 5% in patients with intraocular foreign body compared to 1.7% in patients without it. The statistically significance means that patients with intraocular foreign body have higher risk for progression of endophthalmitis since the foreign body has a high complex of virulence microorganisms that can easily grow in a suitable culture like the human eye.

In this study we found a relationship between low VA and endophthalmitis; all the involved patients had a VA less than1/10. This may be related to the fact that low VA is originated from the more disrupted eye which may have more risk for endophthalmitis due to the entrance of organisms and prolapsed tissue or lens rupture.

In our study, there was no significant difference between two study groups regarding time from trauma to hospitalization and time from trauma to repair. Thompson *et al* found that the eyes that were repaired after 24 hours have a higher incidence of endophthalmitis (Bhagat, Nagori, & Zarbin, 2011). The United States injury registry recommends primary repair after open globe injury as soon as possible preferably within 24 hours (Charles et al., 2003). Delayed primary repair especially after 24 hours is considered to be a risk factor in the absence of intraocular foreign body (Narang et al., 2004). Thompson also found that patients who were repaired in less than 24 hours have lower rate of endophthalmitis (3.5%) than those who were repaired after 24 hours (13.4%).

There were no significant difference between two groups regarding location of trauma in cornea, sclera and sclerocornea in contribution to the higher rate of endophthalmitis. In Faghihi study, corneal injury was a risk factor (Faghihi et al., 2012). This may be contributed to a fact that entrance of microorganisms from any site of eye especially with corneal and lens rupture may have the same risk as the entrance of microorganisms from the posterior site directly to the vitreous body.

The longer duration of hospitalization was contributed to higher incidence of endophthalmitis. This can be explained by the fact that the more severe trauma needs to be more under observation and reasonably has a more risk of endophthalmitis; also more duration of hospital stay may contribute to the high nosocomial infection. The low rate of positive cultures maybe related to the early administration of intravenous antibiotics in the first hours of hospitalization (Narang et al., 2004).

## 5. Conclusion

In our large retrospective analysis, patients with low age, low visual acuity at admission, presence of intraocular foreign body and long duration of hospital stay had a higher risk of endophthalmitis after the repair of the globe, so special care must be taken for these patients by close observation, considering prophylaxis and early treatment.
